# Novel Gels: An Emerging Approach for Delivering of Therapeutic Molecules and Recent Trends

**DOI:** 10.3390/gels8050316

**Published:** 2022-05-19

**Authors:** Trideva K. Sastri, Vishal N. Gupta, Souvik Chakraborty, Sharadha Madhusudhan, Hitesh Kumar, Pallavi Chand, Vikas Jain, Balamuralidhara Veeranna, Devegowda V. Gowda

**Affiliations:** Department of Pharmaceutics, JSS College of Pharmacy, JSS Academy of Higher Education & Research, Sri Shivarathreeshwara Nagar, Mysuru 570015, India; trideva.k@gmail.com (T.K.S.); souvik93pharmacist@gmail.com (S.C.); msharadha1996@gmail.com (S.M.); hitesh.sahu1921@gmail.com (H.K.); pallavichand1990@gmail.com (P.C.); vikasjain@jssuni.edu.in (V.J.); baligowda@jssuni.edu.in (B.V.); dvgowda@jssuni.edu.in (D.V.G.)

**Keywords:** hydrogels, in situ gels, emulsion gels, microgels, nanogels, vesicular gels

## Abstract

Gels are semisolid, homogeneous systems with continuous or discrete therapeutic molecules in a suitable lipophilic or hydrophilic three-dimensional network base. Innovative gel systems possess multipurpose applications in cosmetics, food, pharmaceuticals, biotechnology, and so forth. Formulating a gel-based delivery system is simple and the delivery system enables the release of loaded therapeutic molecules. Furthermore, it facilitates the delivery of molecules via various routes as these gel-based systems offer proximal surface contact between a loaded therapeutic molecule and an absorption site. In the past decade, researchers have potentially explored and established a significant understanding of gel-based delivery systems for drug delivery. Subsequently, they have enabled the prospects of developing novel gel-based systems that illicit drug release by specific biological or external stimuli, such as temperature, pH, enzymes, ultrasound, antigens, etc. These systems are considered smart gels for their broad applications. This review reflects the significant role of advanced gel-based delivery systems for various therapeutic benefits. This detailed discussion is focused on strategies for the formulation of different novel gel-based systems, as well as it highlights the current research trends of these systems and patented technologies.

## 1. Introduction

**Figure 1:**
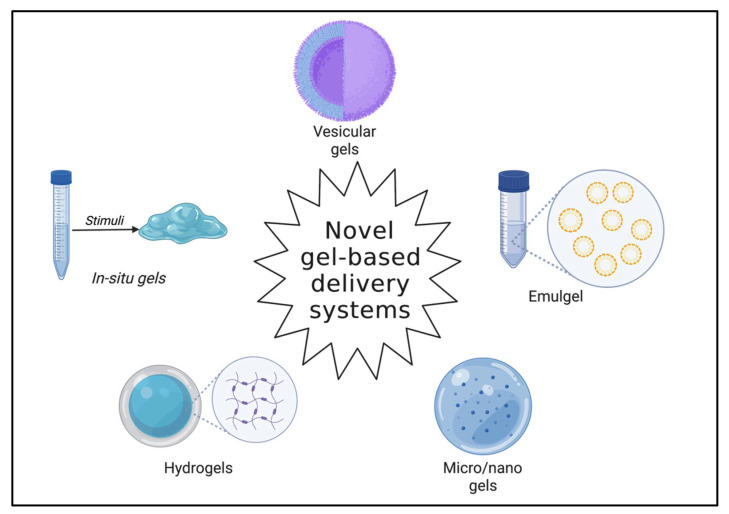
Common novel gel-based delivery systems (Created with BioRender.com accessed on 28 April 2022).

In recent years, novel drug delivery systems have proven very adept at delivering therapeutic molecules with site-specific and localized effects. Additionally, these systems facilitate drug release at desired rates and simultaneously lower the undesired effects [1]. Gels are three-dimensional, semi-solid systems consisting of polymeric matrices. These behave in the same way as solid systems; however, they consist of relatively higher liquid components than solid dispersions [2,3]. Gel systems comprise long, arbitrary chains, albeit with reversible links at precise points. These systems comprise minimum two components and are fundamentally coherent colloidal dispersion systems [4]. The system components, namely the dispersion medium and the dispersed constituent, are uniformly scattered throughout the system. Gels are usually transparent or translucent in appearance entailing higher amounts of solvent [5]. When a suitable solvent is employed, the gelling agents entangle to form a three-dimensional colloidal network that confines fluid movement by entrapment and achieves immobilization of solvent molecules [6]. The network governs the viscoelastic properties of the gel system by developing endurance against deformation. In other words, the thixotropic behavior is contributed by the matrix’s structure [7]. Gels are prepared mainly by fusion technique or by employing gelling agents. Gel-based systems can be alienated into two categories, organogels and hydrogels, based on the physical state of the gelling agent dispersion [8]. Dispersible colloids and water-soluble components constitute hydrogels, while lipophilic oleaginous components are employed in organogels [9]. The systems are further classified into xerogels or aqueous gels based on the nature of the solvents. Xerogels are solid gels with a minimum solvent concentration obtained mainly by solvent evaporation, thereby attaining a gel network [10,11]. However, the gel state can be reinstated by incorporating an imbibing agent that swells the matrix. Novel gels are capable of controlled and sustained release of loaded therapeutic molecules. Figure 1 portrays common novel gel-based delivery systems [12]. Smart gels can be developed which respond to biological and external stimuli, such as temperature, pH, chemical, enzymes, electrical, light, antigens, etc. These systems are highly instrumental in lowering undesired effects and are biodegradable and biocompatible [13]. High drug loading can be achieved. Their size (nanogels) expedites high drug accumulation at the tissue level and enables stealth systems by evading phagocytic cells [14,15,16]. Their distinctive surface properties enable passive and active targeting. This review underlines the advances in gel-based delivery systems, their developments, and a current update in the delivery of therapeutic molecules.

## 2. Advances in Novel Gel-Based Delivery Systems

**Figure 2:**
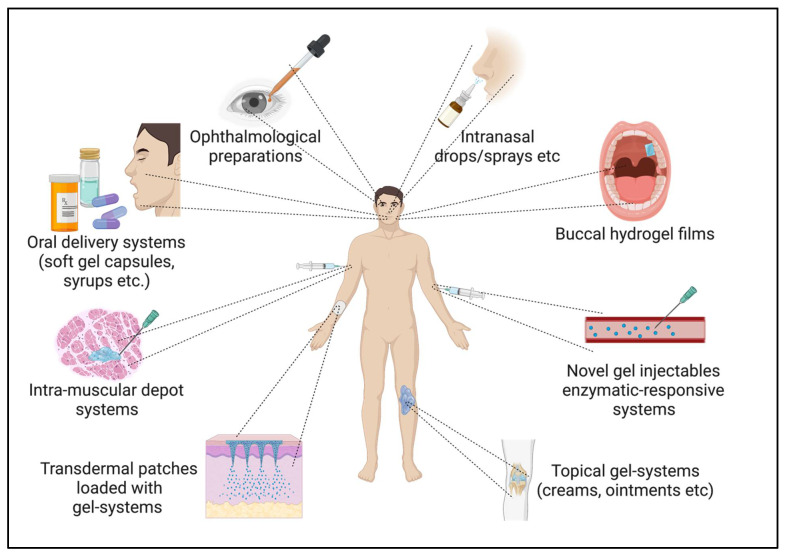
Potential delivery routes for novel gel-based delivery system. (Created with BioRender.com accessed on 28 April 2022).

Novel gel-based drug delivery systems are classified by the nature of their structural network and by their response to stimuli [15]. The former is either a chemically aligned gel network or a physically aligned gel network system. At the same time, the latter category entails responsive, intelligent gel systems that imbibe solvents and swell on exposure to stimuli, such as temperature, pH, chemical, enzymes, electrical, light, antigens, etc. [17,18]. Novel gel systems are evaluated for rigorous characterizations to understand their efficacy as delivery systems. The most commonly employed evaluation parameters comprise swelling capacity, size and morphology, rheological properties, surface charge, etc. [19]. Further, they are scrutinized for physical appearance for compliance, physical state, homogeneity, and phase separation to understand their stability, extrudability, and spreading coefficient is significant for topical gels, as well as bioadhesive strength is a vital element for mucoadhesive gels [20]. Drug content, permeability, and release play a substantial role in any drug delivery system. The International Council on Harmonisation (ICH) (Geneva, Switzerland) dictates the stability guidelines. The gels are subjected to various stress conditions and later scrutinized for drug content, release, and entrapment efficiency to assess their compliance [21]. Figure 2 illustrates various potential delivery routes for novel gel-based delivery systems. 

### 2.1. Intelligent Hydrogels

A three-dimensional network of hydrophilic polymers composes of hydrogels that inherently imbibe water and maintain the system’s integrity [22]. These systems are one of the most versatile delivery systems among novel gels. Researchers have comprehensively developed several intelligent hydrogels that precisely retort to numerous physical stimuli such as temperature, light, electric fields, pressure, sound, and magnetic fields in recent years. Furthermore, stimuli pertain to pH, ions, enzymes, etc. These systems are beneficial for formulating controlled delivery systems [23,24,25]. 

Temperature-responsive hydrogels are triggered by a precisely established temperature range. These hydrogels are formulated with polymers that are capable of temperature-triggered phase transitions [26]. Regular polymers exhibit higher solubility with an increase in temperature; however, the polymers employed in temperature-responsive systems, such as poly (N, N-diethylacrylamide), poly (tertramethyleneether glycol), poly(N-isopropylacrylamide), and others, possess lower critical solution temperatures. Polymers with lower critical solution temperatures tend to shrink with the increase in temperature; these hydrogels are known as negative temperature responsive [22]. Poly(acrylic acid) and polyacrylamide polymers inherently imbibe at higher temperatures and shrink at lower temperatures; hence, hydrogels prepared from these polymers are positive temperature responsive. However, tetronics and pluronics are applied to formulate thermally reversible gels [27]. 

Electrical signal-responsive hydrogels endure swelling and contracting when subjected to an electric field. The demerit of some systems is that due to the charge orientation, one side swells while the other contracts, thus, comprising the stability [28]. Polyelectrolytes, such as poly(2-acrylamido-2-methylpropane sulphonic acid-co-n-butlymethacrylate), are usually employed to formulate these systems [29,30].

pH-responsive hydrogels tend to release or accept protons depending on the pH of the site. Researchers have studied these systems extensively and reported encouraging results. Poly(N,N’-diethylaminoethylmethacrylate) is ionized at a low pH, unlike poly(acrylic acid), which ionizes at a higher pH. However, polycations tend to swell less at neutral pH [31,32].

In enzyme-responsive systems, a suitable enzyme is considered to trigger a release or deliver at a precisely targeted site where the enzyme is operational at a specific temperature or pH [33]. Enzyme-responsive hydrogels are usually prepared from cellulose and other suitable polymers that facilitate the macromolecular networks and function in a controlled environment [34]. The most explored enzymatic stimuli-responsive system consists of a triggerable agent (usually a polymer or a lipid) into which a therapeutic molecule is incorporated. Indeed, this active agent is sensitive to swelling or degradation when it reaches the target site. Some reported enzymes include protease- and glycosidase-based catalyzed enzymatic reactions [35].

Other intelligent systems include light-responsive hydrogels that are functional in ophthalmic delivery systems. These systems are responsive to light and other stimuli, including pressure, thrombin, antigen, and so forth [36,37,38]. 

### 2.2. In Situ Gels

Over the years, in situ gels have exhibited tremendous benefits in controlled drug delivery systems and emerged as a significant intelligent drug delivery technology [39]. These incredible systems remain in a liquid state at room temperature and achieve a sol-gel state when exposed to any biological environments, such as altered pH and temperature. In other words, in situ gelling is a spontaneous gelation process at a specific site post-administration [40]. 

This system accommodates numerous routes of administration, namely ocular, oral, intranasal, vaginal, rectal, depot system, etc. In situ systems have proven their benefits, including prolonged residence time at the site of application. As a result, there is a marked reduction in dosage regimen. Good thixotropic properties expedite the flexibility for formulation development. Rapid absorption and onset can be easily achieved. As a result, the therapeutic benefits can be achieved at lower doses with minimal side effects. Besides, they expedite systemic circulation, avoiding localized hepatic circulation, and targeting can be accomplished [41,42]. 

In situ gel systems principally work on stimuli such as temperature alteration (chitosan, poloxamer), ion activation (sodium alginate), pH changes (carbopol), solvent exchange, and environmental factors. The gels are ideally dependent on physical or chemical mechanisms. The physical mechanism constitutes of imbibing liquids, mainly water and diffusion, and absorbing water by gel polymers in site-specific locations. While diffusion entails the solvent penetration from the polymer solution to the neighboring tissues, the polymer solidifies. Temperature–responsive, pH-responsive, enzymatic cross-linking, and ionic cross-linking are effective mechanisms that govern the precipitation of solids in gel systems [43]. 

The pH-responsive systems include fewer polymers such as cellulose acetate phthalate, carbopol, pseudolatexes, polyethylene glycol, and polymethacrilicacid. The temperature-responsive systems primarily form gels with temperature variations and they include polymers such as pluronics, chitosan, xyloglucans etc. [44,45]. Enzymatic cross-linking is governed by natural biological enzymes. The rate of gel formation is proportional to the enzyme concentration. Insulin delivery was studied with a smart stimuli-responsive delivery system and exhibited positive outcomes, e.g., in a study reported by Podual et.al., where the glucose oxidase enzyme was employed to facilitate the release [46,47,48,49]. In ionic cross-linking, different ions dictate the phase transition of polymers. Gellan gum, carrageenan, and alginic acid are a few ion-responsive polymers. Several natural polymers are available in nature, such as carrageenan, which transform to a gel state on exposure to ions. Gellan gum is predominantly available as Gelrite (commercially available). It is an anionic polysaccharide that instinctively forms a gel in the presence of Mg^++^, Na^+^, K^+^, and Ca^++^. Electromagnetic radiation is applied to facilitate in situ gels in photo-polymerization techniques [50,51]. This is achieved by injecting reactive macromers or a solution with initiators and monomers into the desired tissue site. To initiate photo-polymerization, specific polymerizable functional groups and acrylate or similar macromers that undergo dissociation in the presence of a photo-initiator are subjected to radiation. In ultraviolet photo-polymerization, a ketone, such as 2,2 dimethoxy-2-phenyl acetophenone, is used as the initiator, while camphorquinone and ethyl eosin initiators are used in visible light systems [52,53,54,55]. 

### 2.3. Emulsion Gels

An emulgel is an amalgamation of gel technology and emulsions. This system offers controlled release, especially in topical formulations, as the therapeutic molecules are loaded in a dual delivery system emulsion and a gel core. The fusion of these dual delivery systems overcomes the demerits of these conventional systems, such as stability and drug loading [56]. Emulgels are prepared by incorporating gelling agents in the continuous (usually water) emulsion phase. Compared to other gels, these systems facilitate higher entrapment efficiency, desired thixotropic behavior, and better patient compliance. The formulation of emulgels is achieved by loading the drug-loaded emulsion into a pre-gel and applying a shear to achieve a homogenous gel system [57]. Gelling agents, emulsifiers, water, and penetration enhancers (primarily for topical formulations) are the fundamental components of emulgels. Polyethylene glycol, tweens, spans, and so forth are utilized as emulsifiers, while carbopols, including Hydroxypropyl methylcellulose (HPMC), are used as gelling agents. Menthol, oleic acid, etc., are mostly effective penetration enhancers. Emulgels are amphiphilic and enable the load of both hydrophilic and lipophilic moieties. Furthermore, the bioavailability of specific molecules was enhanced by lowering the globule size to a micron (µm). Microemulsions are isotropic, clearer, and stable systems. As a result, the increased effective surface facilitates a higher bioavailability [58]. These systems are proven better than emulgels due to the significant increase in the penetration of topical formulations and better compliance [59]. 

### 2.4. Microgels

Microgels are described as gels that have a size range in microns (µm). In contrast to typical gels, these systems have cross-linking structures in microns (µm) and are colloidal dispersions [60]. The molecular arrangements are different in these systems, in comparison to regular gels. Electric charge, polymer–water bonding, and cross-link density are a few factors that underlie the swelling capacity of these systems [61]. The internal forces impart steadiness to the microgels. Similar to microgels, stimuli-responsive methods yield novelty. The colloidal nature of microgels augments characteristics such as controlled delivery of therapeutic molecules, high responsiveness to stimuli, extrudability, mass transport, and high therapeutic efficacy with minimal adversity [62]. The microgels can be created by reducing the size of macrogels by applying a shear or by employing monomers or polymers. The basic underlying principles for formulating are emulsification, where a pregel is prepared in the oil phase, followed by polymerization, yielding microgels. In nucleation, the adjacent particles (in the solvent) initiate the nucleation process and deliver homogeneous microgels [63]. No external forces are required. In contrast, complexation, where complexes are formed in water, can be achieved by adding two mild polymers to water. Another peculiar method can be adopted for developing microgels by the mere addition of polyelectrolytes, which are oppositely charged and at diluted concentrations yield colloidal dispersion [64]. 

### 2.5. Nanogels 

Nanogels are mostly hydrogel polymeric network dispersions with particles lying in the nanometer range (nm). Nanogels allow chemical modifications to facilitate ligand targeting and triggered release. This system is a blend of colloidal dispersions and cross-linking networks of polymers [65]. Nanogels are capable of intracellular delivery with enhanced cellular concentrations without significant cellular toxicity [66]. Other advantages include rapid swelling, sharp responsiveness to stimuli, high bioavailability, accommodating more therapeutic moieties, avoiding renal clearance, and remaining undetected by opsonins. Besides, nanogels have excellent stability and accommodate highly lipophilic moieties [67]. Distinctly, nanogels can be prepared by chemical cross-linking (emulsion polymerization), pulse radiolysis, and photopolymerization. In the first method, the cross-linking agent, monomers, surfactant, and water are added to an organic phase. Later, this organic phase is irradiated and purified. In pulse radiolysis, ionizing radiation is irradiated onto polymers in suitable solvents to promote internal structural reorientations of polymer radicles to yield nanogels. In the photopolymerization technique, monomers are exposed to UV radiation. Initiators, cross-linking agents, or surfactants are not required for this method; hence, pure nanogels are produced [68,69].

Similarly, other procedures include heterogenous controlled radical polymerization. Recently, developed techniques, including reversible addition–fragmentation chain transfer and atom transfer radical polymerization, have been instrumental in developing polymer-conjugate systems. Chemical cross-linking is more commonly employed to formulate varied nanogels and is further classified into Michael addition reactions, carbodiimide coupling, and free radical polymerization [70]. Hydrophilic monomers are polymerized in the presence of varied cross linkers to yield synthetic nanogels. Self-assembly of polymers includes an aggregation of the hydrophilic polymers by electrostatic interactions, hydrophobic bonds, or hydrogen bonding in an aqueous medium. These systems accommodate large molecules; thus, they are effective in incorporating macromolecules, such as proteins and peptides [71]. 

### 2.6. Vesicular Gels

Vesicular gels are composed of carrier systems deliberate with amphiphilic molecules comprising lipids, surfactants, and co-polymers [72]. The carrier system consists of a hydrophilic core within an amphiphilic bilayer [73]. Vesicular drug delivery has been on the rise in recent studies for its versatility and broad application in drug delivery, cosmetics, etc. Vesicular gels have exhibited significant results in topical drug delivery. A hydrogel matrix can be incorporated with preformed vesicles in a simple technique [74,75]. 

#### 2.6.1. Liposomal Gels 

Liposomes are a very effective and successful novel delivery system. One significant advantage of these systems is that they possess biosimilar structures with desirable properties [76]. Their amphiphilic properties enable the incorporation of both lipophilic and hydrophilic therapeutic molecules. These carriers are biodegradable, exhibit no toxicity, and support localization and site-specific release. They are capable of infiltrating several bio-obstacles otherwise difficult to any conventional systems [77,78]. However, often the topical applications are limited due to rheological constraints. Traditionally, liposomes can be formulated by the standard technique of lipid film hydration. Other techniques include the French pressure cell method and solvent injection methods. The addition of a gelling agent in the aqueous phase incorporates liposomes into the gel system. Carbopols, xanthan gum, poloxamers, gellan gum, polyvinyl alcohol, and others are explored as gelling agents [79].

#### 2.6.2. Niosomal Gels

Niosomes are similar to the liposomal system; however, these are prepared from non-ionic surfactants. This vesicular system resolves the instability of traditional liposomes due to phospholipids [80]. The bilayer, depending on the preparation technique, yields unilamellar or multilamellar niosomes. The system’s integrity relies on the surfactant’s chemical composition and on the hydrophilic–lipophilic balance (HLB) value. Primarily, proniosomes, emulsion niosomes, and aspasomes are different types of niosomes. Proniosomes are preliminary niosomes which are devoid of any aqueous phase [81]. These are reconstituted with a suitable buffer to yield niosomes. Proniosomes are advantageous over niosomes regarding dose dumping and stability. Niosomal gels comprise a therapeutic moiety, surfactant, and cholesterol that yield vesicles, which are later loaded into a gel base. However, broad applications of these systems are restricted to achieving controlled release, stealth systemic circulations, and enhanced stability [82,83]. 

#### 2.6.3. Transferosome Gels

Transferosomes are improved versions of liposomes. These systems overcome various shortcomings of other vesicular systems, including aggregation, dose dumping, and poor permeability [84]. Similar to liposomes, these systems also possess an aqueous core enclosed by a lipid layer. However, these bilayers are modified with edge activators, which enables flexibility. Usually span 80, tween 80, and sodium cholate are proven effective edge activators. Phospholipids, edge activators, alcohol, and hydrating media (buffers) constitute transferosomes [85]. Hydrocolloids are incorporated into buffers to yield transferosome gels. The delivery of proteins and peptides, such as insulin, has shown promising results when incorporated into the transferosome gel system [85,86,87]. 

## 3. Novel Gel-Based Delivery Approaches for Delivering Therapeutic Molecules—Recent Trends

### 3.1. Hydrogel Systems 

Various nanocarrier systems have been utilized in drug delivery applications for different diseases. The number of cancer patients is increasing day by day worldwide. However, the therapy for cancer treatment is still adapted as a conventional method, i.e., surgery and radiotherapy followed by chemotherapy. There are several limitations of conventional chemotherapy, which produces long- or short-term side effects and adverse effects. Such chemotherapy delivery to cancer patients has low bioavailability due to poor solubility issues, resulting in adverse effects on the biological systems. Hence, researchers are finding a better way to deliver chemotherapeutics with enhanced bioavailability to resolve these problems safely. The hydrogel drug delivery system is one of the carrier systems that can deliver hydrophilic chemotherapeutics agents and hydrophobic drugs in a sustained and controlled manner. Generally, hydrogels are hydrated in nature and possess self-shrinking and self-swelling characteristics in different biological conditions [88]. Their 3D structural properties enable the efficient encapsulation of chemotherapeutic agents into their internal structure, which protects the drug from degradation either in storage or enzymatically during circulation in biological systems. The advantage of the hydrogel drug delivery system in cancer therapy is that nanogels can be modified according to the response to the cell or the tumor microenvironments [89].

Hydrogels can be functionalized by targeting ligands for enhanced, prolonged, and specific drug delivery, making it a safer carrier system. In general, hydrogels could achieve high drug delivery efficiency in cancer therapy with conformational changes and degradation under specific conditions, such as temperature, pH, redox, and ultrasound [90]. 

The second advantage is that hydrogel synthesis can be done as per the demand of the drug delivery to particular tumor microenvironments. Internal and external stimuli have been utilized to design hydrogels for the delivery of chemotherapeutic drugs to manage cancer. The thermo-sensitive stimuli hydrogels are the most common used hydrogels. These hydrogels are usually in a gel state at room temperature and are attributed to a low critical solution temperature. Once it is administered into the body, it will change its form into a solution due to the cellular temperature. Usually, poly(N-isopropylacrylamide) or elastin-like polypeptides have been used in thermo-sensitive hydrogels recently [91,92].

Moreover, thermo-sensitive hydrogel in situ sites avoid the accumulation of chemotherapeutic drugs in the liver or spleen, which overcomes the biosafety limitations of drugs [93]. The chemotherapeutic agent, 7-ethyl-10-hydroxycamptothecin or SN-38, has a lower solubility in drug delivery applications. Bai et al. developed a thermo-sensitive liposomal system, which showed a better antitumor effect and reduced systematic toxicities in in vivo models at the same dose of the pure drug [94]. Similarly, alginate-based thermo-sensitive gels enhanced cisplatin’s in vivo antitumoral effects through an in situ injection. They spurred the tumor growth up to 95% compared to the control group, along with an increased prolonged survival rate of the animals [95]. Furthermore, in another study, the interaction of ligand–receptor with the hydrogel enhances transcorneal permeability and precorneal retention of the drug activity. Upon topical instillation, dexamethasone and Arg-gly-asp supramolecular hydrogel increased the transcorneal permeability in rabbits’ eyes to treat ocular inflammation [96]. The development of a copolymer from a mono-functional polymer exhibits a good sol-gel transition phase, thereby enhancing water solubility to prolong the mucoadhesive system. The effect of silsesquioxane thermo-responsive hydrogel of FK506 improved drug solubility, biocompatibility, and prolonged retention time by enhancing drug efficacy in a murine dry eye model [97].

These hydrogels can be used for single-drug therapy and combinatorial therapy by co-loading with other chemotherapeutics drugs [98,99]. For instance, Doxorubicin (DOX), IL-2, and IFN-g were delivered by a poly (g-ethyl-L glutamate)-poly (ethylene glycol)-poly(g-ethyl-L-glutamate) (PELG-PEG-PELG) hydrogel. The prepared hydrogel system showed long-term sustained drug release behavior for more than three weeks. The combination therapy enhanced the antitumor effect against B16F10 melanoma cells by inducing cell apoptosis and cell cycle arrest in the G2/S phase. The nanocarriers have not shown any systematic side effects in the xenograft mice model, suggesting an effective and promising approach to drug delivery in melanoma therapies [98]. 

### 3.2. Thermosensitive Hydrogels

Likewise, the thermo-sensitive poly(3-caprolactone)-10R5-PCL hydrogels, co-loaded with tannic acid and oxaplatin to manage colorectal cancer, restricted the CT26 colon cancer growth in a mice model. They improved the survival time of the animals (11). Moreover, the co-delivery of gemcitabine and cisplatin through the PDLLA-PEG-PDLLA hydrogels synergistically improved the anti-cancer efficacy against pancreatic cancer with sustained drug release. The dual drug-loaded hydrogels exhibited superior antitumor effects in the xenograft model than in single-drug therapies [100]. Similarly, when the thermo-sensitive stimuli hydrogel was utilized to co-deliver paclitaxel (PTX) and temozolomide (TMZ), it produced a synergistic effect against glioblastoma cells. The in vivo studies suggested that the combination therapy potentially reduced tumor growth and sustained drug release in mice brains for one month with no apparent side effects [101]. 

Chitosan-based thermo-sensitive hydrogels, including several polyols, have gained a critical identity that transforms to a hydrogel form upon contact with body temperature from a solution state. A study indicated that a hydrogel formulation that employs a green synthesis approach with chitosan, genepin, and poloxamer 407 has proved to sustain drug release. Brinzolamide-loaded nanostructured lipid carrier was entrapped into a hydrogel matrix using a hot-melt emulsification and sonication method that showed a sustained drug release for a longer duration (24 h) than marketed eye drops (8 h) in the management of glaucoma [102]. Moxifloxacin hydrochloride thermosensitive gel was formulated using chitosan-β-glycerophosphate to advance the ocular delivery. The drug release profile of the formulation was shown to be delivered in a sustained pattern and at a slower rate with a release of 53% in 1 h and 83.3% in 8 h due to the hydrogel’s polymeric network, whereas a 75.6% release was identified from the drug solution. Hence, the formulated hydrogel was stated to be biodegradable, safe, and with a more significant drug loading to the administration site to manage bacterial infections [103]. The quaternized chitosan achieved a better swelling property as a therapeutic carrier. The thermo-sensitive transparent quaternized chitosan hydrogel was utilized to release timolol maleate in the management of glaucoma. Hemolysis and cytotoxicity profiles showed good biocompatibility. The in vitro release pattern of timolol maleate from the hydrogel showed a burst release initially and a linear release for one week, showing a sustained pattern. Hence, quaternized chitosan has a promising ability to sustain drug release in the anti-glaucoma model [104]. Similarly, chitosan has been an extensively used polysaccharide to prolong precorneal retention and corneal permeability, though it is poorly soluble in physiological solvents. Therefore, derivates of chitosan (glycol chitosan) have shown superior aqueous solubility in a broad pH range and are employed in ocular drug delivery systems, such as hydrogels, nanoparticles, and films. Hydrogel films for the topical ocular delivery of dexamethasone and levofloxacin were fabricated by utilizing many oxidation degrees of oxidized hyaluronic acid and glycol chitosan. The formulation showed potent activity in decreasing bacterial growth in different strains. Additionally, the formulation downregulated in vitro anti-inflammatory activities. Overall, the formulated hydrogel film would serve as a treatment for endophthalmitis with minimal corneal irritation and biocompatibility [105]. In another study, a combination of carbon dots and thermo-sensitive hydrogels was evaluated for their in vitro cellular toxicity after effectively delivering diclofenac sodium to an eye. The in vitro release showed characteristic biphasic release of the drug. Cellular toxicity studies revealed that the formulation has a better cytocompatibility with CD44 targeting and serves as a novel way for ocular delivery of drugs [106].

### 3.3. Light Stimuli Hydrogels

Furthermore, light stimuli hydrogels enhanced drug release from hydrogels. The mechanism of photosensitive hydrogels is that they can undergo structural and conformational changes under radiation, ultraviolet, and visible light sources and achieve sol-gel transition [107,108]. Photo stimuli hydrogels have been utilized in the past few years. For example, Fourniols and his co-worker developed a polyethylene glycol dimethacrylate-based photo-polymerized hydrogel for the local and sustained delivery of TMZ in the management of glioblastoma [109]. Similarly, azobenzene, α-cyclodextrin-functionalized hyaluronic acid, and gold nano-bipyramids-mesoporous silica nanoparticles-conjugated polymer-based in situ injectable hydrogels loaded with DOX and stimulated under near-infrared (NIR) radiation potentially improved the drug localization of drug into the nuclei of the tumor cells [110]. Another example of a NIR stimuli hydrogel was designed by Qui et al. in 2018. They synthesized DOX and black phosphorus-loaded agarose-based hydrogels. The drug release was enhanced after exposure to the light intensity of NIR, which therapeutically increased the anti-cancer efficacy for in vivo experiments [111].

### 3.4. pH Stimuli Hydrogels

Another most commonly used hydrogel for cancer therapy is the pH stimuli hydrogel. Due to the diverse microenvironments of tumor cells, the pH of the extracellular matrix of the tumor is in the range of 5.8–7.2 and the lysosomal or intracellular matrix pH is usually 5.5, which is acidic compared to normal cells (pH 7.4) [112,113]. Hence, both intracellular and extracellular acidic conditions stimulated the hydrogels for degradation and drug release at the tumor site. The pH-sensitivity could have been achieved by protonating the polymers’ ionizable moiety or acid-cleavable bond break [114]. 

Usually, the pH stimuli hydrogels act as prodrugs, inactive in normal biological pH (7.4), but once they reach the tumor site, upon the release of the drug, they change to their active chemotherapeutic form, producing its effect. The FER-8 peptide hydrogel loaded with PTX exhibited high drug encapsulation and self-assembly of the peptide at pH 7.4. The hydrogel was stimulated by the acidic conditions of the tumor microenvironment. The PTX-loaded hydrogel significantly accumulated the drug in the tumor site and showed sustained and prolonged retention of PTX through an intratumoral injection. This hydrogel showed enhanced tumor inhibition [115]. The polyacrylic acid-based pH stimuli hydrogel for DOX delivery triggered the specific site of drug delivery with a less acidic microenvironment. The hydrogels showed improved pharmacokinetics and drug accumulation in a mice xenograft model with significant tumor growth regression and lowered adverse and side effects [116]. Various hydrogels have received attention in the last few years due to their biocompatibility, biodegradability, and low toxicity. The hydrogel-based drug delivery of chemotherapeutic agents has been attended in recent years [27]. Most chemotherapeutic drugs are associated with lower solubility. Hence, efficient drug delivery and significant therapeutic effects cannot be achieved by a lower dose and the involvement of a higher dose in cancer management includes side effects. Therefore, the hydrogel system is the emerging nanocarrier for the delivery of chemotherapeutic agents. Some recent significant research related to novel gel-based systems is shown in Table 1.

## 4. Descriptive Patents Established for Novel Gel-Based Delivery Systems

Various publications illustrated the efficacy of novel gels over the last decades. The current section explains the established patents of novel gels. In patent 20210338211, aptamer and hydrogels cross-linked with DNAzyme are used for colorimetric identification of analytes in body fluids through an ocular device [150]. Patent 2021120395 tells that in situ hydrogels provide extended drug release by delivering to a tissue, usually agents with low water solubility [151]. Patent W/O/2021/113515 explains that hydrogels composed of Gelatin–hydroxyphenylpropionic acid (gelatin-HPA), hyaluronic acid–tyramine (HA-Tyr), catalyzer, cross-linker, or other combinations can treat ocular disorders [152]. Patent 20210069496 describes polymeric formulations, including hydrogels formed by a UV cross-linking method. Here, the hydrogels act as a nasal stimulator that stimulates the lacrimal glands to mimic the production of tears electronically and to manage dry eye syndromes [153]. Patent WO/2021/038279 tells of the invention related to ion-exchange polymeric hydrogels for ocular treatment [154].

Patent 202121042889 talks about etoricoxib, which is formulated as a nanosponge hydrogel for the management of arthritis by the method of emulsion solvent diffusion using a polymeric organic solvent, ethylcellulose eudragit, and an aqueous phase. The formulated nanosponges were evaluated for differential scanning calorimetry (DSC), Fourier-transform infrared spectroscopy (FTIR), polydispersity index (PDI), scanning electron microscopy (SEM), zeta potential, drug content, entrapment efficiency, viscosity, spreadability, in vitro diffusion, irritation test, and in vivo antiarthritic effect. The synthesized formulation proved to be effective as a novel way for managing arthritic pain [155]. In patent 202031000910, a cross-linked protein matrix hydrogel was prepared for topical application in skin regeneration and wound healing [156].

Conductive hydrogels with an adhesiveness method of preparation were invented and discussed in patent 112442194. The process involves dopamine modification of carbon nanotubes and grafting to saccharides, followed by acrylamide mixing and formation of hydrogels in the presence of an initiator and a cross-linking agent. Hydrogel structure, electrical conductivity, adhesiveness, and biocompatibility can be improved by dispersing the modified carbon nanotubes in an aqueous solution to form hydrogen bonds and cross-link with the supramolecules of the hydrogel. Hence, conductive hydrogels can be used in biomedical fields, as well as for human body monitoring and electronic skin, etc. [157].

Patent 20210023121 discloses thrombin-responsive hydrogels for prolonged heparin delivery for auto-anticoagulant regulation in a controlled feedback mechanism. The formulated microneedle, containing a patch, can activate the thrombin and release heparin to avoid blood coagulation. The insertion of a microneedle patch containing hydrogel regulates blood coagulation sustainably in response to thrombin without leakage [158].

Patent 20210393780 discloses the effectiveness of thermo-sensitive polymer–protein-based hydrogels in the field of cancer therapeutics. The invention is enriched by photosensitizers, dyes, photothermal agents, and drugs. Hence, the invention proved to be less expensive, highly effective, and thermosensitive, resulting in a sustained drug release for targeted delivery [159]. Patent WO/2021/174021 describes a degradable hydrogel system for immunotherapy with an extended-release pattern of an anti-cancer drug linked with a hydrogel matrix synergistically for cancer treatment [160]. Patent 9758/CHENP/2012 explains self-assembling peptides and their use in hydrogels for the adhesion, proliferation, differentiation of neural stem cells, and their auto-healing properties. They are reported to be non-toxic in central nervous systems, as well as to avoid bleeding and have faster nervous regeneration [161]. Patent 20140286865 explores di-block co-polypeptide synthetic hydrogels in the central nervous system [162].

## 5. Conclusions 

In this review, the authors have focused on recent trends in novel gel-based drug delivery systems and their applications. In recent times, these novel systems have exhibited proficient delivery of multiple therapeutic moieties and expressed desired properties and functions, such as selective targeting. The systems offer abundant benefits compared to conventional drug delivery approaches including controlled drug release, high drug loading, biocompatibility and biodegradability, and enriching patient compliance and comfort. The responsive gel technology is significant in formulating intelligent delivery systems; these systems respond to stimuli such as pH, temperature, enzymes, and so forth. Hence, these systems are site-specific and facilitate the controlled release of therapeutic molecules. Although, fundamentally, these systems have proven capabilities for effective drug delivery, there is a scope to explore new polymers to fabricate novel gels; therefore, the currently employed components can be modified. Furthermore, recent studies revealed that employing plant extracts to develop novel delivery systems has enabled the development of various drug delivery systems with non-toxic procedures. The formulation of substances of natural origin has advantages in various magnitudes on the environment. Over the years, the evolution of green chemistry has provided more eco-friendly procedures resulting in minor harm to nature. Current findings suggest promising results for green synthesised delivery systems over conventional systems. Green technology does not require common harmful chemicals. Instead, this technology uses biological and biocompatible reagents. Besides, reports suggest that green technology delivery systems have better stability than traditional methods. Formulations developed with green technology employing plant extracts and biomaterials, such as proteins or peptides, yielded non-toxic and highly biocompatible systems; thus, they have resolved the most concerning issue with traditional delivery systems, i.e., toxicity. Green technology will play a significant role in formulating novel delivery systems. However, we require further understanding of the development of systems with green technology.

## Figures and Tables

**Table 1:** Recent published research for novel gel-based delivery systems.

Sno.	Types of Hydrogels	Composition	Drug Used	Disease	References
**1**	**Thermo-stimuli Hydrogel**	7-ethyl-10-hydroxycamptothecin (SN-38) liposomal hydrogel	SN-38	Hepatocellular carcinoma	[94]
**2**		alginate nanogel co-loaded with cisplatin and gold nanoparticles	cisplatin	colorectal cancer	[95]
**3**		poly(γ-ethyl-L-glutamate)-poly(ethylene glycol)-poly(γ-ethyl-L-glutamate) (PELG-PEG-PELG) hydrogel	DOX/IL-2/IFN-γ	melanoma	[98]
**4**		PHB-b-PDMAEMA	paclitaxel and Temozolomide	glioblastoma	[99]
**5**		poly(3-caprolactone) (PCL)-10R5-PCL (PCLR) hydrogel	tannic acid/ oxaliplatin	colorectal cancer	[117]
**6**		Caprolactone-Polyethylene Glycol	Silibinin	melanoma	[118]
**7**		α-Cyclodextrin co-polymeric PEGylated iron oxide-based hydrogels	PTX/DOX	breast cancer	[119]
**8**		β- cyclodextrin complexed glycol chitosan hydrogel	PTX	Ovarian Cancer	[120]
**9**		mPEG-b-PELG	CA4P and cisplatin	colorectal cancer	[121]
**10**		Pluronic F127, Pluronic F68, and Hydroxy Propyl Methyl Cellulose.	Itraconazole	Fungal Keratitis	[122]
**11**		Sulfobutylether-β-cyclodextrin (SBE-β-CD)	Ketoconazole	Fungal Keratitis	[123]
**12**		Poloxamers (P407 and P188), Carbopol-93	Dipivefrin hydrochloride	Intraocular pressure	[124]
**13**		Triacetin, Transcutol-P, Poloxamer 407, Poloxamer188	Acyclovir	Ocular viral infections	[125]
**14**		Poloxamer 407, disodium EDTA	Chlorhexidine digluconate	*Acanthamoeba* keratitis	[126]
**15**		poloxamers, hyaluronic acid (HA), beta-lapachone (β Lap),	beta-lapachone (β Lap)	Restoring the synovial fluid	[127]
**16**		Poloxamers, D—(+)-GlcN hydrochloride, papain,	Glucosamine (GlcN)	controlling inflammation and promoting cartilage re-generation	[128]
**17**		Poloxamer, hyaluronic	Sulforaphane (SFN	SFN intra-articular release for OA treatment	[129]
**18**	**Photosensitive Hydrogels**	azobenzene and α-cyclodextrin-functionalized hyaluronic acid with gold nanobipyramids	DOX	human epidermal keratinocyte	[110]
**19**		poly(N-isopropylacrylamide) hydrogel	Bortezomib and DOX	osteoblast	[130]
**20**		Agarose based hydrogel	black phosphorus and DOX	breast cancer	[111]
**21**		poly(*N*-phenylglycine)- poly (ethylene glycol) co-polymeric hydrogel	Cisplatin	breast cancer	[131]
**23**	**pH-stimuli Hydrogels**	poly (acrylic acid) complexed with stabilized amorphous calcium carbonate	DOX	hepatocarcinoma	[116]
**24**		amphiphilic hyaluronan (HA)-and cystamin-pyrenyl	Organoiridium (III)	lung cancer	[132]
**25**		Graphene oxide, L-arginine	5-fluorouracil	breast cancer	[133]
**26**		FER-8 peptide	PTX	hepatocarcinoma	[115]
**27**		Dibenzaldehyde, poly (ethylene glycol)	DOX	hepatocarcinoma	[50]
**28**	**Redox- stimuli Hydrogels**	dextrin nanogel	DOX	Breast Cancer	[134]
**29**		Polydopamine, poly (ethylene glycol)	DOX	breast cancer	[135]
**30**		poly (ethylene glycol) monomethacrylate	Vorinostat and etoposide	cervical cancer	[136]
**31**		polyglycerol nanogel	DOX	cervical cancer	[137]
**33**		N-Isopropylacrylamide, Methacrylic acid, Benzalkonium chloride and poly (sulfobetaine methacrylate)	DOX and Indocyanine green	hepatocarcinoma	[138]
**34**	**Magnetism-Responsive Hydrogels**	ferromagnetic vortex-domain iron oxide, chitosan and poly (ethylene glycol)	DOX	breast cancer	[139]
**35**		methacrylic acid, ethylene glycol dimethacrylate, 2,2′-azobisisobutyronitrile and glycidyl methacrylate	sunitinib	cervical cancer, breast cancer and Human Thyroid Tumor	[140]
**36**		paramagnetic fullerene, DNA and Hyaluronic Acid	DOX	hepatocarcinoma	[141]
**37**	**Proniosomal gel**	Surfactant, lecithin and cholesterol	Curcumin	Ocular Inflammation	[142]
**38**	**Liposomal gel**	Lecithin: cholesterol, Carbopol 934	Travoprost	Glaucoma and ocular hypertension	[143]
**39**	**Injectable hydrogel**	horseradish peroxidase (HRP) and H2O, chitosan, hyaluronic acid (HA)	Dextrane Tyramine	cartilage tissue regeneration	[144]
**40**		bone marrow mesenchymal stem cell (MSC) spheroids, short fibre fillers, Kartogenin (KGN)	Celecoxib	cartilage regeneration, and inflammation removal	[145]
**41**		poly (ethylene glycol)-*b*-polythioketal-*b*-poly(ethylene glycol), micelles	dexamethasone acetate	preventing cartilage extracellular matrix degeneration	[146]
**42**		Gelatin, ulbecco’s phosphate buffered saline (DPBS), methacrylic anhydride.	diclofenac sodium	preventing the development of degenerative changes in OA via the synergistical treatment of enhanced lubrication (COF reduction) and sustained drug release (inflammation down-regulation)	[147]
**43**		Hexachlorocyclotriphosphazene, Poly (dichlorophosphazene, Methoxy poly (ethylene glycol),	Triamcinolone acetonide	Effective prevention and long-term anti-OA treatment	[148]
**44**	**Shear—sensitive hydrogels**	Hyaluronic acid, aldehyde groups, amino Groups, HSPC lipid,	Celecoxib	Minimizing shear-induced cartilage damage and inflammation	[149]

## Data Availability

Not applicable.
